# Domestic Pets and Skin Diseases

**Published:** 1883-01

**Authors:** 


Domestic Pets and Skin Diseases.—Dr. Wooster Beach of this city, reports a case of ring worm on the cheek of a child, probably produced by contact with a pet kitten which was diseased.

Two similar cases have been reported in the *Lancet.*

Ringworm is also said to be gotten from horses.

Other contagious diseases may be traced to same kind of source.

Thus Dr. McCall Anderson traces the development of the disease known as favus *(Porrigo favosa)* in human beings to mice suffering from the disease. Cats, which eat the mice, catch the disease and have been known to communicate it to the children who handle them. Fowls have also been known to suffer from it. The danger of allowing children to handle domestic pets which are suffering from skin disease is probably often overlooked, and deserves to be made known more widely than it is at present.

